# Mattering as a Political, Scientific, and Professional Basis for Welfare Services

**DOI:** 10.3389/fpsyg.2021.734630

**Published:** 2021-09-22

**Authors:** Steinar Krokstad

**Affiliations:** ^1^HUNT Research Centre, Department of Public Health and Nursing, Norwegian University of Science and Technology, Trondheim, Norway; ^2^Levanger Hospital, Nord-Trøndelag Hospital Trust, Levanger, Norway

**Keywords:** welfare-to-work, mattering, neoliberalism, cultural psychology, structural disadvantage, victim blaming

## Abstract

The dominant political ideology of recent decades, neoliberalism, have resulted in diminished sense of mattering for several groups in the society, not at least people outside the labor market. This has left its mark on vocational rehabilitation programs in welfare states like Norway. Higher requirements shall be set for benefit recipients, and compulsory work are more often applied. The problem with this policy is that it suggests that benefit recipients have a guilt to make up for and are themselves to blame for the unemployment. However, the majority of people in need for vocational rehabilitation, have had poor living conditions since childhood, and have failed in education and employment for or reasons they have no control over. They often do not feel valued and have a lot of experience with not being able to add value. The problem with blaming the victims, is that it reinforces their sense of worthlessness, and thus reduces their ability to believe that they can contribute with something of value. In this way, the policy becomes counterproductive. Some even respond to these humiliating pressures by becoming more depressive or aggressive. To make vocational rehabilitation programs effective, we must make sure that everyone in need for it feel valued, we must align the political, scientific, and professional basis for welfare service politics thereafter. We must balance adding value to self with the opportunity to adding value to others, work and community. Mattering is suggested as a political, scientific, and professional basis for welfare services.

## Introduction

It is a legitimate goal that as many as possible are in paid work in a modern welfare state, if the job does not harm their health or quality of life. For the majority, being in work contributes to a feeling of value and an opportunity to contribute with something valuable in life (Prilleltensky, 2020). In addition, disability and chronic illness are associated with poverty and social exclusion. Work is one of the main sources of income and social position, but people with disabilities and chronic illnesses have disproportionately low employment rates in many developed economies (Bambra et al., 2005). There are considerable economic and social costs to individuals and society associated with short and long term sickness absence from work (OECD, 2010). In Norway, high employment has been high on the political agenda in the post-war period, and politics has been successful (OECD, 2021).

Neoliberal ideologies, which have been the dominant political ideologies of most Western nations the last decades, have economic growth as a main purpose (Cook, 2012; Kotz, 2017). Economic growth is important for public health development, but in developed countries the distribution of money has a greater impact on public health than gross domestic product (Pickett and Wilkinson, 2015; Rosling et al., 2018). Neoliberalism sees competition as the defining characteristic of human relations. So pervasive has it become that we seldom even recognize it as an ideology. It redefines citizens as consumers, rewards merit and punishes inefficiency (Cook, 2012; Monbiot, 2016). Thus, in neoliberal societies, people outside the labor market have low value because they are not economically productive. This has left its mark on vocational rehabilitation programs in many welfare states (Larner, 2000), also in Norway. Vocational rehabilitation programs is to a larger degree motivated by economic and not humane needs (Cook, 2012). Higher requirements shall be set for benefit recipients, and compulsory work or mandatory activation are more often applied (Dahlberg et al., 2009). Perspectives of cultural psychology may contribute to understand this change in policy with its fundamental attribution error, to overattribute behaviors among social benefit recipients to their personality and underattribute them to the situation or context (Heine, 2011). The problem with the ideology is that it suggests that benefit recipients have a guilt to make up for and are themselves to blame for the unemployment (Krokstad, 2021). Because when the state makes claims against citizens who receive social benefits, it also says that the person in question is not really entitled to the benefit and puts the person’s morals in doubt.

Welfare states establish various programs to support transitions to work for people who are unemployed or underemployed or receive social benefits (Lahey et al., 2019). The literature shows, though, that very few return-to-work programs have proven to be effective. The proportion of participants gaining employment after involvement in one or the other of schemes may be up to 50%. However, few studies are designed using controls. Study designs with controls often shows non-statistical significant effects (Bambra et al., 2005). Another weakness is that most studies follow-up participants over short time periods. It is not necessarily difficult to push people into work in the short term, by spending a lot of resources on individual follow-up and using strong individual economic incentives. But the goal should rather be to get people into long term sustainable employment, with positive impact on their health and quality of life. Because if people not really have health resources to tackle the job they are guided into, there is an imminent danger of deteriorating health, reduced quality of life, new long-term sick leave and a further reduction in the person’s mattering (Cook, 2012).

## What Matters in Welfare-To-Work

Two problems arise when the blame for unemployment is individualized; the structural causes are not addressed, and the measures become ineffective. There is extensive evidence that the large majority of people with health problems and in need for vocational rehabilitation, have had poor living conditions since childhood (Tomasdottir et al., 2015), and have failed in education and employment for structural factors they not have had control over (Dubow et al., 2009). They often do not feel valued and have a lot of experience with not being able to add value to themselves, others, work, or community (Prilleltensky, 2020). Thus, citizens who are included in welfare-to-work programs, have a greater need to matter than most in society.

### Feeling Valued

Mattering consists of feeling valued and adding value. When citizens feel valued, they are appreciated, respected, and recognized. When citizens add value, they are able to make a contribution or make a difference (Elliott et al., 2004). Early in life, survival needs are met by caregivers. The quality of attachment to caregivers is highly influential in many outcomes in life, like educational achievement. Depending on parental emotional availability, children develop different attachment styles that are going to have a lasting impact throughout life (Shaver and Mikulincer, 2012). Attachment security provides a psychological foundation for how people manage their lives (Prilleltensky, 2020). But feeling that you have a value is just as important in adulthood – throughout the life cycle. That is why the first thing citizens in welfare-to-work programs should meet with is appreciation, respectfulness, and recognition. Without the belief that we have value we cannot get out of bed, finish a degree, or manage a job.

### Adding Value

However, being valued is a necessary but insufficient for mattering. To feel like a valuable citizen, to matter, we need skills and opportunities to add value, to contribute to ourselves and others (Elliott et al., 2004; Pancer, 2013). In a modern welfare state, this often means being in paid work. As we grow, we search for new skills, and paths to potentiate our talents if this is possible and supported by the environment. All of us want to make a difference, in our lives, and the lives of others. Several established psychological theories underscore the universal need to add value: self-determination (Ryan and Deci, 2017), self-efficacy (Bandura, 1995), and meaning in life (Prilleltensky, 2020). Competence is important to master the environment and feel effective. But to we need more than formal education, we need to know how to manage ourselves and how to manage other people. Self-efficacy is the belief that we can achieve certain outcomes (Bandura, 1997).

### Social Policy

The historical background of social policies with measurements taken by state to protect workers and the establishment of welfare states, is linked to the human history. The industrial revolution was an economic revolution on one side but increased the social problems on the other side. Seeking solutions to address the poverty and social imbalance, which were caused by the industrialization, social policy tried to make balance between economy and social policies (Esping-Andersen, 2017).

In Norway and other Nordic countries welfare schemes were designed based on social democratic policies in the post-war period (Esping-Andersen, 2017). The aim was to redistribute power, income and play a regulatory role and eliminate negativity in working life. Social security and welfare services were developed. The social policy included social security – compensation for lost earnings, but also accessible health services for all and other measures against unemployment and poverty. In a broad sense, the final target of all these practices was to ensure social peace, social justice, and equality between different groups.

The dominating political ideology the latest years, however, adopted a view that social expenditures hamper economic growth. Ideas about shrinking of welfare states and reduction of its role on social policies gained momentum (Bregman, 2017). With a policy toward shrinkage in the welfare state, the provision of welfare services has also changed. Citizens who were previously considered to need support because they were unable to work for various health and social reasons, were to varying degrees characterized as unprofitable and thus of lower value. The problem with this practice of blaming the victims, is that it reinforces citizens sense of worthlessness, and thus reduces their ability to believe that they can contribute with something of value. Therefore, the policy is in danger of becoming counterproductive. Some even respond to such humiliating pressures by becoming more depressive or even aggressive (Prilleltensky, 2020). Now, in societies marked by this ideological shift, mental illness and associated pain and fatigue have become the dominating public health challenges (Tyrovolas et al., 2020), and the main cause of incapacity for work (Patel et al., 2007).

## Discussion

Welfare states are developing in many countries like in Norway. Politicians are in search for new solutions seen from different ideological points of view. The welfare programs have great support in the population, so politicians are careful to propose downscaling. From this perspective, it is not a real crisis in welfare states, but to reduce the passive expenditures and reduce taxes, the period of benefiting from social benefits is typically shortened, and their conditions are made difficult. The idea is to remove obstacles for economic growth. However, there is a high risk of harming vulnerable citizens based on a failing scientific and professional basis (Prilleltensky, 2020). An OECD report have shown that Norway would have higher economic growth if income were distributed more equitably (OECD, 2015), and countries in the OECD with the most generous social insurance systems have the highest employment rates (Barth et al., 2015). Countries spending more money on welfare have often had higher economic growth during the economic downturn than countries that reduce welfare benefits (Stuckler and Basu, 2013). There are many indications that cutting social benefits will not increase economic growth, but rather contribute to the opposite (Navarro, 1998), and new research now underpins that social benefits are investments, not expenses (Bregman, 2017).

According to the neoliberal ideology, the unemployed are often considered to be of less value to society, as they are not economically active. Thus, higher requirements shall be set for benefit recipients, and compulsory work or mandatory activation are more often applied. This suggests that benefit recipients have a guilt to make up for and are themselves to blame for the unemployment. In this way, their basic need to feel valued and ability to add value is undermined (Prilleltensky, 2020).

To make vocational rehabilitation programs effective and not blame the victims of structural disadvantage, we must make sure that everyone who needs help feel valued, and align the political, scientific, and professional basis for welfare service politics thereafter. We must balance adding value to citizens with the opportunity to adding value to other people and through work to community, to counteract the increasing marginalization of valuable citizens. Mattering is therefore proposed as a political, scientific, and professional basis for nations welfare programs and services.

## Data Availability Statement

The original contributions presented in the study are included in the article/supplementary material, further inquiries can be directed to the corresponding author.

## Author Contributions

SK ideated the structure, analyzed the literature, and wrote the manuscript.

## Conflict of Interest

The author declares that the research was conducted in the absence of any commercial or financial relationships that could be construed as a potential conflict of interest.

## Publisher’s Note

All claims expressed in this article are solely those of the authors and do not necessarily represent those of their affiliated organizations, or those of the publisher, the editors and the reviewers. Any product that may be evaluated in this article, or claim that may be made by its manufacturer, is not guaranteed or endorsed by the publisher.
